# Supplementary material for: Field physical model tests on the mechanism of river blocking by debris flow in the middle reaches of the Dadu River, Southwest China
Source: PLoS One. 2024 Apr 29;19(4):e0298076. doi: 10.1371/journal.pone.0298076 (PMC11057723; doi:10.1371/journal.pone.0298076)
Supplement: Attachment — Submitted filename: Review comments.docx [file pone.0298076.s001.docx]

Review comments

This interesting study investigated the dynamic processes and river-blocking modes of debris flow using field physical experiments. Although this is a very physical-intensive work, the paper is poorly written and lacks in-depth discussion.

1. Abstract is poorly organized with many wrong expressions and lacks a clear knowledge gap.

L24-25, a wrong sentence. L29-30, the logic is wrong.

L33-36, the implication is badly summarized.

2. This paper is poorly written that some paragraphs of the paper are in a lack of readability because of too redundant information and language issues. I suggest that the authors focus on the style/language and to ask a professional editor to go over the whole text.
3. Introduction would benefit from a critical review of relevant literature, identifying gaps in our knowledge and justifying their approach to the topic.

L45-L53, listing many historic stories is not good.

L57-69, these introductory content should appear in the Study area and Material.

L91-103, directly listing many references is very bad.

4. Figures 1-5 introduces the background of debris flow in the Dadu River, but this study focus on the field experiments. The relation between actual condition and experiment results should be separated. I think Figures 1-5 should be combined in one figures.
5. Figures 8b, 10b, 12b have low resolution.
6. In Figures 9, 11, 13, 14, fitting all data using the linear function is not appropriate.
7. Table 1 and Table 2 is useless.
8. Discussion is missing.
9. Conclusion almost repeats the Abstract.
