# Supplementary material for: Field physical model tests on the mechanism of river blocking by debris flow in the middle reaches of the Dadu River, Southwest China
Source: PLoS One. 2024 Apr 29;19(4):e0298076. doi: 10.1371/journal.pone.0298076 (PMC11057723; doi:10.1371/journal.pone.0298076)
Supplement: Attachment — Submitted filename: Response to Reviewers.doc [file pone.0298076.s002.doc]

**PLOS ONE**

**Response to the Comments from Reviewers**

Manuscript number: PONE-D-23-02409

Full title: Field physical model tests on the mechanism of river blocking by debris flow in the middle reaches of the Dadu River, Southwest China

Article type: Research paper

Authors: Zhi Song, Yanni Chen, Gang Fan

We would like to thank the reviewer and the Editor for their comments and constructive suggestions. We have considered these comments and revised the manuscript accordingly. Listed below please find our responses to the editor’s and reviewer’ comments.

**Response to the Comments from reviewer #1**

**Q**1. Abstract is poorly organized with many wrong expressions and lacks a clear knowledge gap.

L24-25, a wrong sentence. L29-30, the logic is wrong. L33-36, the implication is badly summarized.

**Response**: Thanks for your comment. The abovementioned sentences are revised by the authors.

**Q**2. This paper is poorly written that some paragraphs of the paper are in a lack of readability because of too redundant information and language issues. I suggest that the authors focus on the style/language and to ask a professional editor to go over the whole text.

**Response**: Thanks for your comment. The manuscript has been polished by a native English speaker. Attached is the editing certification of this manuscript.

**Q**3. Introduction would benefit from a critical review of relevant literature, identifying gaps in our knowledge and justifying their approach to the topic.

L45-L53, listing many historic stories is not good.

L57-69, these introductory content should appear in the Study area and Material.

L91-103, directly listing many references is very bad.

**Response**: Thanks for your comment. The manuscript has been revised. The introductory content about the middle reaches of the Dadu River has been moved to “the Study area and Material”. Additional, the citation of the references is revised.

**Q**4. Figures 1-5 introduces the background of debris flow in the Dadu River, but this study focus on the field experiments. The relation between actual condition and experiment results should be separated. I think Figures 1-5 should be combined in one figures.

**Response**: Thanks for your comment. As you mentioned, 5 figures are adopted in the manuscript to introduce the basis information of the study area, it seems superfluous. Accordingly, only 2 figures are retained to show the debris flows in the study area, i.e., “Figure 1 Typical debris flow cases in the study area” and “Figure 2 Distribution of debris flows and (b) debris flow density in the study area”, other figures are deleted. Since Figure 1 and Figure 2 include too many information, it is hard to merge these two figures into one figure, hence the Figure 1 and Figure 2 are retained in the revised manuscript.

**Q**5. Figures 8b, 10b, 12b have low resolution.

**Response**: Thanks for your comment. Figures 8b, 10b, 12b have been redrawn and improved.

**Q**6. In Figures 9, 11, 13, 14, fitting all data using the linear function is not appropriate.

**Response**: Thanks for your comment. According to the authors’ attempts, the linear function is the optimal one with the maximum correlation coefficient. Accordingly, a sentence is added in the revised manuscript to state this issue.

**Q**7. Table 1 and Table 2 are useless.

**Response**: Thanks for your comment. Table 1 and Table 2 have been deleted in the manuscript, accordingly, some hydrological information of study area is added in the manuscript, please see Lines 71-73.

**Q**8. Discussion is missing.

**Response**: Thanks for your comment. A discussion is added in the revised manuscript.

**Q**9. Conclusion almost repeats the Abstract.

**Response**: Thanks for your comment. The conclusion is revised.

**Response to the Comments from reviewer #2**

**Q**1. Please add necessary references in some places, such as lines 48-53.

**Response**: Thanks for your comment. The citation of the references in the manuscript is revised.

**Q**2. In the introduction, the summary and description of previous work, that is, the work of predecessors, lacks organization, indicating a lack of logic in the entire introduction. On the basis of summarizing previous work, deficiencies in previous research should be identified. Of course, this deficiency needs to be related to the work of this manuscript. This can lead to a more reasonable introduction to the work of this manuscript. Therefore, I suggest the author to reorganize the introduction.

**Response**: Thanks for your comment. The introduction has been revised according to the comments. The revised manuscript has emphasized the research gap between the current studies and this study.

**Q**3. This manuscript lacks a discussion section, which is the most important part of an academic paper. It is recommended that the author add it. The achievements and limitations of this study should be discussed from various aspects such as data, methods, and results. In addition, it is also necessary to provide several research or work prospects.

**Response**: Thanks for your comment. A discussion is added in the revised manuscript to discuss the data, method and the research results, and also the potential prospects on the future research.

**Q**4. The language and expression of this manuscript are not very good, it is recommended to polish the manuscript. For example, in the abstract, the meaning of this sentence is not clear and needs to be modified –“The particle size and density of sand are more likely to block the river with mixed-flow-type river-blocking mode”.

**Response**: Thanks for your comment. The whole manuscript was checked and polished carefully.
