# Supplementary material for: Field physical model tests on the mechanism of river blocking by debris flow in the middle reaches of the Dadu River, Southwest China
Source: PLoS One. 2024 Apr 29;19(4):e0298076. doi: 10.1371/journal.pone.0298076 (PMC11057723; doi:10.1371/journal.pone.0298076)
Supplement: Attachment — Submitted filename: Comments on PONE-D-23-02409R1.docx [file pone.0298076.s003.docx]

Comments on PONE-D-23-02409R1

This study carried out field physical model tests to study the mechanism of river blocking by debris flow in the middle reaches of the Dadu River. It tried to reveal the dynamic processes of river blocking by debris flows and proposed three typical river-blocking modes of debris flow. By reviewing the revised manuscript, all the comments from previous reviewers have been fully responded and the manuscript has been revised properly. This revised manuscript could be reconsidered for publication with minor revisions. The comments on this version are as follows.

(1) The research gap is suggested to enhance in the manuscript, to address the significant importance to the disaster prevention and mitigation in the middle section of the Dadu River.

(2) Determining of geometric scale is a key factor in the design of physical model tests, so the authors are suggested to state the reason why the geometric scale was determined to be 1: 500 and its effect on the test results.

(3) The key factors that determine the specific river-blocking modes of debris flow should be discussed in the Discussion.
