# Supplementary material for: Field physical model tests on the mechanism of river blocking by debris flow in the middle reaches of the Dadu River, Southwest China
Source: PLoS One. 2024 Apr 29;19(4):e0298076. doi: 10.1371/journal.pone.0298076 (PMC11057723; doi:10.1371/journal.pone.0298076)
Supplement: Attachment — Submitted filename: Response to Reviewers-R2.doc [file pone.0298076.s004.doc]

**PLOS ONE**

**Response to the Comments from Reviewers**

Manuscript number: PONE-D-23-02409

Full title: Field physical model tests on the mechanism of river blocking by debris flow in the middle reaches of the Dadu River, Southwest China

Article type: Research paper

Authors: Zhi Song, Yunxin Zhan, Yanni Chen, Gang Fan

We would like to thank the reviewer and the Editor for their comments and constructive suggestions. We have considered these comments and revised the manuscript accordingly. Listed below please find our responses to the editor’s and reviewer’ comments.

**Response to the Comments from reviewer #1**

**Q**1. The research gap is suggested to enhance in the manuscript, to address the significant importance to the disaster prevention and mitigation in the middle section of the Dadu River.

**Response**: Thanks for your comment. The significant importance of this study has been addressed in the revised manuscript, please see **Lines 107-111**.

**Q**2. Determining of geometric scale is a key factor in the design of physical model tests, so the authors are suggested to state the reason why the geometric scale was determined to be 1: 500 and its effect on the test results.

**Response**: Thanks for your comment. According to the limitations of the field test site and the capacity of the test equipment, the geometric scale of this model test was finally determined to be 1:500. Accordingly, the reason why the geometric scale was determined to be 1: 500 is stated in the revised manuscript. Please see **Lines 241-246**.

**Q**3. The key factors that determine the specific river-blocking modes of debris flow should be discussed in the Discussion.

**Response**: Thanks for your comment. The discussion was revised, please see **Lines 519-538**.

**Response to the Comments from reviewer #2**

**Q**1. The author has made revisions to the manuscript, resulting in an improvement in its quality. Regarding the discussion section, however, the current discussion section is too shallow and it is difficult to imagine the discussion section of an academic paper without references, which is unacceptable. Suggest the authors rewrite the discussion section. The achievements and limitations of this study should be discussed from various aspects such as data, methods, and results In addition, it is also necessary to provide multiple research or work perspectives.

**Response**: Thanks for your comment. The discussion was revised, please see **Lines 519-538**.
